# The *N*-Substituted-4-Methylbenzenesulphonyl Hydrazone Inhibits Angiogenesis in Zebrafish *Tg(fli1: EGFP)* Model

**DOI:** 10.3390/ph15111308

**Published:** 2022-10-23

**Authors:** Monika Gawrońska-Grzywacz, Iwona Piątkowska-Chmiel, Łukasz Popiołek, Mariola Herbet, Jarosław Dudka

**Affiliations:** 1 Department of Toxicology, Faculty of Pharmacy, Medical University of Lublin, 8B Jaczewskiego Street, 20-090 Lublin, Poland; 2 Department of Organic Chemistry, Faculty of Pharmacy, Medical University of Lublin, 4A Chodźki Street, 20-093 Lublin, Poland

**Keywords:** angiogenesis, zebrafish, *Tg(fli1: EGFP)*, sulphonyl hydrazone, FET

## Abstract

One of the most important therapies of malignant neoplasms, which are the second cause of death worldwide, is focused on the inhibition of pathological angiogenesis within the tumor. Therefore, the searching for the efficacious and relatively inexpensive small-molecule inhibitors of this process is essential. In this research, the anti-angiogenic potential of *N*-substituted-4-methylbenzenesulphonyl hydrazone, possessing antiproliferative activity against cancer cells, was tested. For this purpose, an intersegmental vessel (ISV) angiogenesis assay was performed using 6 hpf (hours post fertilization), 12 hpf and 24 hpf embryos of zebrafish transgenic strain, *Tg(fli1: EGFP)*. They were incubated with different concentrations of tested molecule and after 24 h the development of intersegmental vessels of the trunk was analysed. In turn, the acute toxicity study in the zebrafish model was mainly conducted on strain AB, using the OECD-approved and recommended fish embryo acute toxicity test (FET) procedure. The results showed the moderate toxicity of *N*-[(3-chloro-4-methoxyphenyl)methylidene]-4-methylbenzenesulphonohydrazide in above-mentioned model with the LC_50_ value calculated at 23.04 mg/L. Moreover, newly synthesized molecule demonstrated the anti-angiogenic potential proved in *Tg(fli1: EGFP)* zebrafish model, which may be promising for the therapy of neoplastic tumors as well as other diseases related to pathological angiogenesis, such as age-related macular degeneration and diabetic retinopathy.

## 1. Introduction

Zebrafish (*Danio rerio)* is one of the most frequently used fish species in scientific research including diverse toxicological, pharmacological, behavioural and genetic studies [1,2,3]. It is also an extremely beneficial model in the study of diseases and pathologies affecting blood vessels. We are talking particularly about transgenic *Tg(fli1: EGFP)* individuals, which express the enhanced green fluorescence protein in the vascular endothelium. It allows for careful observation of the process of an angiogenesis or pathological conditions within the blood vessels [4]. Angiogenesis is the creation of new capillaries from already existing blood vessels, and it is regulated by pro- and anti-angiogenic factors. An imbalance between these substances can lead to the development of cardiovascular diseases. A fairly common anomaly in the vascular structure is neovascularization of the eye, accompanying diseases such as macular degeneration or diabetic retinopathy [5]. In neoplastic diseases, angiogenesis is out of control and plays a key role in tumor growth and metastasis [6]. The process of neoplastic angiogenesis consists in the branching and growth of already existing vessels. It starts with the local degradation of the basal membrane in the vessel wall from the tumor side under the influence of a pro-angiogenic factor. Neoplastic angiogenesis enables good blood supply to the tumor that promotes metastasis, as cancer cells more easily enter the bloodstream, and subsequently even to distant organs and tissues. Therefore, the control of angiogenesis processes is particularly important in limiting the growth and metastasis of neoplasms [6].

Over the years, for pre-selection of potentially active compounds various models for assessing angiogenesis, both in vitro and ex vivo, have been developed. The choice of the endothelial cell cultures is associated with a loss of complexity of the whole structure, e.g., retina, or physiological limitations (lack of blood flow). In turn, although ex vivo models allow the preservation of tissue complexity, they do not allow to map the pathological angiogenic process of mature vessels. It occurs in diseases such as age-related macular degeneration or diabetic retinopathy [7]. Therefore, an animal model based on fast-maturing, small animals is desirable. These conditions are met by *Danio rerio*. It shows an extraordinary speed in the formation of vessels, which makes small molecules screening feasible. The influence of the tested compounds on the process of angiogenesis can be observed in 24-h cycles because this is the adequate time to complete a single loop of blood circulation from the moment of fertilization. In the 20-th hour, the formation of intersegmental vessels of the trunk takes place, forming a characteristic ladder almost along the entire length of the fish’s body [8].

Sulphonyl hydrazones similarly like hydrazide-hydrazones are organic compounds characterized by the presence of an azomethine group. Their chemical structure is an excellent starting point for the synthesis of various heterocyclic scaffolds [9]. These substances are also distinguished by numerous properties which are important from the medical and pharmaceutical point of view. The biological activity of compounds from sulphonyl hydrazone and hydrazide-hydrazone group described so far includes antimicrobial, anti-tuberculosis, anti-HIV, anticonvulsant, analgesic, anti-inflammatory, antioxidant, antidepressant, and most importantly, from the point of view of this work–anticancer activity [9,10,11,12,13,14,15,16,17]. In our recently published study, the newly-synthesized benzenesulphonyl hydrazones demonstrated the antiproliferative potential towards three human cancer cell lines: renal cell carcinoma (769-P), liver cancer (HepG2) and lung squamous cell carcinoma (H2170). However, the 769-P cell line proved to be particularly the most sensitive to their action and among novel compounds *N*-[(3-chloro-4-methoxyphenyl)methylidene]-4-methylbenzenesulphonohydrazide tested in the presented study showed one of the most significant cytotoxicity towards these cells with an estimated IC_50_ of 26.38 µM. The aforementioned derivative inhibited tumor cell viability selectively without affecting the reference Vero cells. Selectivity index calculated as the ratio between the IC_50_ values in Vero and 769-P cell lines was 55.26 [18]. The treatment of choice for renal cell carcinoma is surgery to remove the affected kidney, however the anti-angiogenic therapy is also very important, since classical chemotherapy is ineffective. Particularly, the immunotherapy with interferon α-2a or, alternatively, treatment with interferon α and bevacizumab, an anti-VEGF monoclonal antibody, can be used to delay the onset of tumor progression. In the second line of treatment, the patients are given inhibitors of tyrosine kinases, mainly the VEGF receptor (VEGFR), and in the third, everolimus—an inhibitor of serine-threonine kinase mTOR (mammalian target of rapamycin kinase)—which reduces VEGF concentration and inhibits the growth and multiplication of tumor cells [19,20].

The present study was aimed at the assessment of the in vivo toxicity profile and anti-angiogenic action of promising *N*-substitued-4-methylbenzenesulphonyl hydrazone possessing potential medical applicability associated with its antiproliferative activity, particularly against renal cell carcinoma [18]. In this type of cancer, the anti-angiogenic therapy is highly recommended. Systemic therapy involves bevacizumab first, followed by axitinib, sorafenib, or pazopanib, which are VEGFR inhibitors. However, in the case of favourable and intermediate prognosis, the therapy starts with receptor tyrosine kinase inhibitors: sunitinib or pazopanib, and in case of failure, axitinib is implemented [19,20]. The effective therapy of pathological angiogenesis, which is one of the main factors in the growth of solid tumors, is considered to be the most important challenges of modern oncology. This requires searching for new, small-molecule inhibitors of this process, because the most commonly used monoclonal antibodies are mostly inefficient and, in addition, they generate very high costs of therapy. For the aforementioned purposes, in the presented research, the zebrafish model was successfully used. Acute toxicity tests according to FET assay guidelines and the intersegmental vessel (ISV) angiogenesis assay were conducted. Zebrafish embryos of both strains, wild-type AB and transgenic *Tg(fli1: EGFP)* were used in the experiments focused on the toxicity assessment. However, transgenic zebrafish embryos, which express the enhanced green fluorescence protein (EGFP) in vascular endothelium, were used to provide insight into the process of angiogenesis.

## 2. Results

### 2.1. Chemistry

The *N*-[(3-chloro-4-methoxyphenyl)methylidene]-4-methylbenzenesulphonohydrazide was synthesized by the condensation reaction of 4-methylbenzenesulphonohydrazide and an appropriate substituted aromatic aldehyde (3-chloro-4-methoxybenzaldehyde), as previously described [18]. The obtained derivative is a stable solid at room temperature and its spectral data (^1^H NMR, ^13^C NMR) is in full agreement with the proposed structure [18].

### 2.2. Toxicity Assessment

#### 2.2.1. Calculation of the LC_50_ Values

After 96 h of exposure of zebrafish larvae of both strains (AB and *Tg(fli1: EGFP)*) to the test substance, the mortality was noted and the LC_50_ values were calculated. Embryo was considered as dead if at least one of toxicity endpoints was observed: embryo coagulation, lack of somite formation, lack of detachment of the tail from the yolk sac and/or lack of heartbeat. The LC_50_ values were estimated at 23.04 mg/L (67.97 µM) for wild-type AB strain and 79.06 mg/L (233.35 µM) for transgenic *Tg(fli1: EGFP)* strain. Negative and solvent control, on the contrary to the positive control, had no embryotoxic effects on zebrafish larvae of both tested strains.

#### 2.2.2. Developmental Malformations

**Figure 1:**
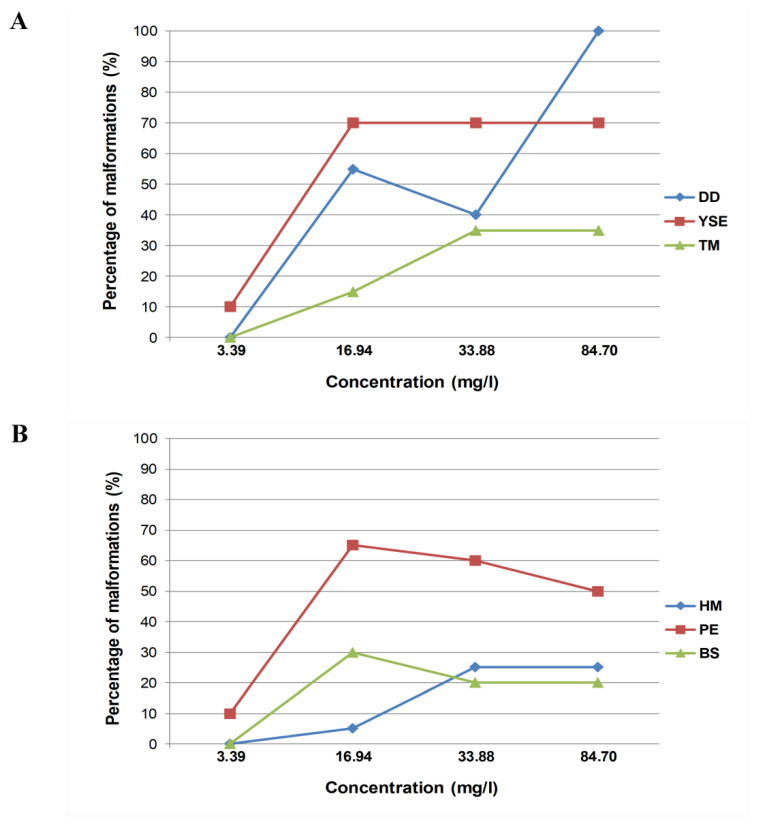
The percentage of developmental malformations in 96 hpf zebrafish larvae (wild-type AB strain) exposed to different concentrations of *N*-[(3-chloro-4-methoxyphenyl)methylidene]-4-methylbenzenesulphonohydrazide. Concentrations: 3.39 mg/L = 10 µM; 16.94 mg/L = 50 µM; 33.88 mg/L = 100 µM; 84.70 mg/L = 250 µM. (**A**): Malformations: DD–developmental delay, YSE—yolk sac edema, TM—tail malformation. (**B**): Malformations: HM–head malformation, PE—pericardium edema, BS–bent spine.

In AB strain of *Danio rerio*, yolk sac edema (YSE) as well as pericardium edema (PE) were the most common malformations observed in larvae after 96 h of exposure. However, after incubation with the concentration of 3.39 mg/L (10 µM) the incidences of YSE and PE were at the very low level (10%). They raised when the concentration of molecule increased to 16.94 mg/L (50 µM) but remain constant at higher concentrations (33.88 mg/L i.e., 100 µM and 84.70 mg/L i.e., 250 µM). The maximum percentages recorded at zebrafish larvae were 70 and 65, respectively (Figure 1A,B). Similarly, the incidences of bent spine are comparable at a range of concentrations 16.94–84.70 mg/L (50–250 µM). This abnormality as well as a developmental delay were not present after exposure to the compound at the lowest concentration tested (3.39 mg/L, 10 µM) (Figure 1A,B). The developmental delay was noted in max. 100% of zebrafish larvae after the incubation with the highest concentration (84.70 mg/L, 250 µM) (Figure 1B). At the lowest concentration tested (3.39 mg/L, 10 µM), no head and tail malformations were found. These abnormalities achieved the highest percentages at 25 and 35, respectively, for 33.88 mg/L (100 µM) and they did not increase when the concentration increased to 84.70 mg/L (250 µM) (Figure 1A,B). 

### 2.3. Zebrafish Larvae Cardiac Rhythm Measurement

**Figure 2:**
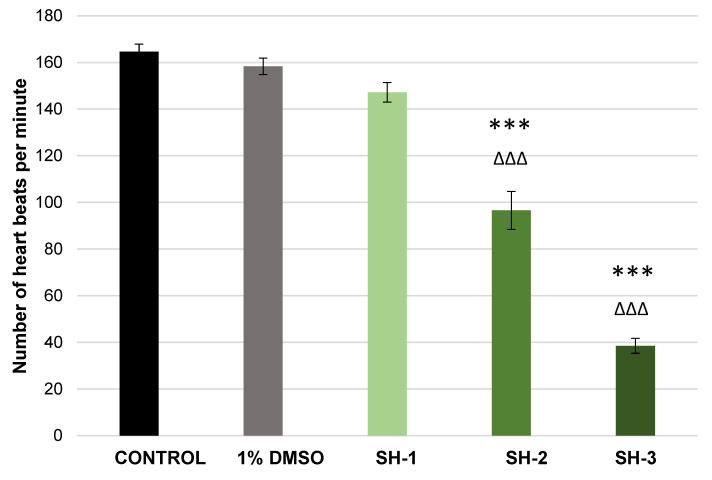
The number of heart beats per minute of zebrafish larvae (wild-type AB strain) after 96-h exposure to control solutions and *N*-[(3-chloro-4-methoxyphenyl)methylidene]-4-methylbenzenesulphonohydrazide (**SH**) at the following concentrations: **1**—16.94 mg/L (50 µM), **2**—33.88 mg/L (100 µM), **3**—84.70 mg/L (250 µM) (*** *p* < 0.001 vs. negative control; ∆∆∆ *p* < 0.001 vs. 1% DMSO).

Figure 2 shows the results of the measurement of heart rate of zebrafish larvae (AB strain) after 96-h exposure to control solutions (negative control and solvent control) and tested compound-*N*-[(3-chloro-4-methoxyphenyl)methylidene]-4-methylbenzenesulphonohydrazide at the following concentrations: 16.94 mg/L (50 µM), 33.88 mg/L (100 µM) and 84.70 mg/L (250 µM). Tested molecule at a concentration of 16.94 mg/L (50 µM) did not affect cardiac rhythm of zebrafish larvae. However, when the embryos were exposed to the concentrations of 33.88 mg/L (100 µM) and 84.70 mg/L (250 µM) for 96 h, very statistically significant (F _[2.42]_ = 68.402; *p* < 0.001) reductions in heart beats per minute were observed when compared to both, negative control (E3) and solvent control (1% DMSO).

### 2.4. Angiogenesis Assay

**Figure 3:**
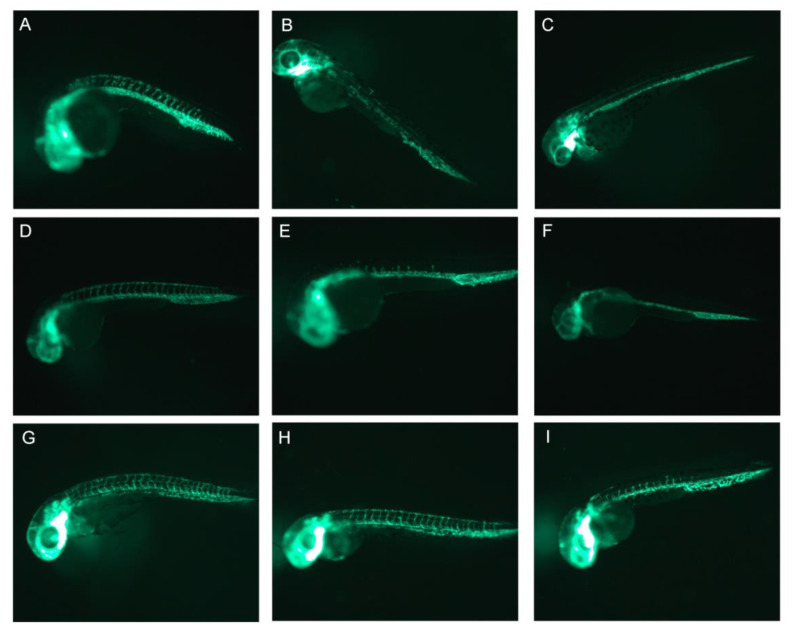
The anti-angiogenic effect observed in *Tg(fli1: EGFP)* zebrafish embryos exposed 6 hpf (**B**,**C**), 12 hpf (**E**,**F**) and 24 hpf (**H**,**I**) to sorafenib at 1.16 mg/L (2.5 µM) (**B**,**E**,**H**) and 2.32 mg/L (5 µM) (**C**,**F**,**I**) vs respective negative controls (6 hpf-(**A**), 12 hpf-(**D**), 24 hpf-(**G**)) (24-h exposure; (**A**–**C**) 63× magnification, (**D**–**I**) 50× magnification).

In the groups of embryos exposed to both doses of sorafenib (1.16 mg/L i.e., 2.5 µM and 2.32 mg/L i.e., 5 µM), the anomalies in the structure of the vessels were observed. The 100% of embryos exposed to the concentration of 1.16 mg/L (2.5 µM) at 6 and 12 h after fertilization (Figure 3B,E) showed the presence of only single intersegmental vessels, which were shorter than those in the negative control (Figure 3A,D). The dorsal longitudinal anastomotic vessel did not develop (Figure 3B,E). In contrast, all the embryos exposed at 24 h after fertilization did not demonstrate any deformation or deficiencies in the vascular structure (Figure 3H). The 100% of 6 and 12 hpf embryos incubated with sorafenib at a concentration of 2.32 mg/L (5 µM) (Figure 3C,F) exhibited a complete absence of intersegmental and dorsal longitudinal anastomotic vessels. In all the 24 hpf embryos exposed to this concentration, vessel defects can be observed, especially around the tail (Figure 3I). The intersegmental vessels were visibly shorter than the normal ones, and the dorsal longitudinal anastomotic vessel was poorly formed (Figure 3I). Embryos’ viability of ≥90% was observed after an exposure for 24 h and any other malformations were not observed.

**Figure 4:**
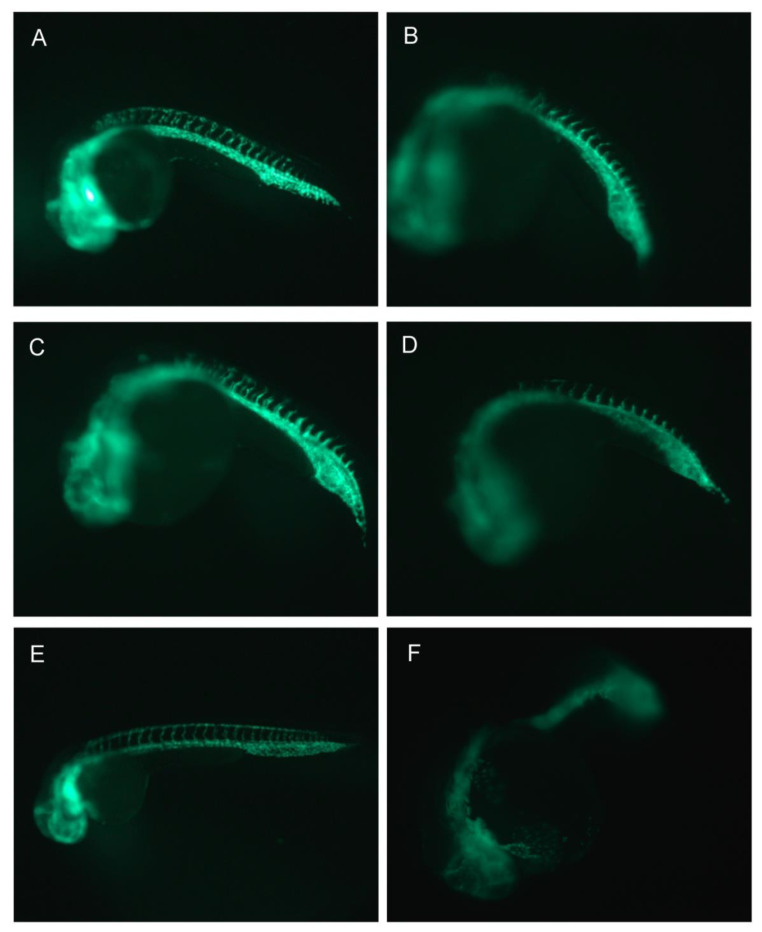
The anti-angiogenic effect observed in *Tg(fli1: EGFP)* zebrafish embryos exposed 6 hpf (**A**–**D**) and 12 hpf (**E**,**F**) to *N*-[(3-chloro-4-methoxyphenyl)methylidene]-4-methylbenzenesulphonohydrazide at a concentration of 15.81 mg/L (46.67 µM) vs respective negative controls (6 hpf-(**A**), 12 hpf –(**E**)) (24-h exposure; ((**A**–**D**) 63× magnification, (**E**,**F**) 50× magnification).

The *N*-[(3-chloro-4-methoxyphenyl)methylidene]-4-methylbenzenesulphonohydrazide proved to have an influence on the angiogenesis process tested in zebrafish embryos of *Tg(fli1: EGFP)* strain. Depending on the concentration, it induces incomplete growth of intersegmental blood vessels between the dorsal aorta and the dorsal longitudinal anastomotic vessel. This effect is only evident when the highest concentration of the compound is used in the 6 hpf and 12 hpf embryo groups. Using a concentration of 15.81 mg/L (46.67 µM; 1/5 LC_50_), the percentage of embryos with vascular defects is 40% when exposed 6 h after fertilization (6 hpf), and for 12 hpf embryos it is 24%. The 6 hpf embryos were characterized by a poorly developed dorsal longitudinal anastomotic vessel and the intersegmental vessels were shorter than normal (Figure 4). However, in 12 hpf embryos exposed, the intersegmental vessels and the dorsal longitudinal anastomotic vessel were not formed at all (Figure 4).

The viability of embryos exposed to the substance at the concentrations for 24 h was ≥80%. No other malformations were observed.

## 3. Discussion

The World Health Organization indicates that malignant neoplasms are the second cause of death worldwide, after cardiovascular system diseases, and their effective treatment is still a challenge for modern medicine [21]. One of the modern treatment strategies is the inhibition of pathological angiogenesis within the tumor. Angiogenesis, the formation of new blood vessels, plays a key role in many processes in the living body, including physiological. However, in the process of neoplasm it gets out of control and is of great importance for the survival and local invasion of neoplastic cells as well as their metastasis [6,8]. The monoclonal antibodies are most often used in therapy what generate very high costs, which requires searching for inhibitors of angiogenesis among newly synthesized compounds.

The review of scientific literature proved a wide range of potential applications and beneficial therapeutic effects of compounds containing hydrazone moiety, like hydrazide-hydrazones or sulphonyl hydrazones [11,13,15,16,18]. The results of much research, including those carried out by our research group, indicated that molecules containing hydrazone moiety are cytotoxic and cytostatic towards neoplastic cells [18,22,23]. The most essential problem that should be solved in the studies focused on the new derivatives, is the creation and synthesis of compound that will be both effective in therapy and non-toxic. Therefore, in vivo toxicity profile and anti-angiogenic potential of promising benzenesulphonyl hydrazone derivative possessing antiproliferative activity, particularly against renal cell carcinoma, were assessed in our study using zebrafish models (AB and *Tg(fli1: EGFP)*) [18]. Every 24 h, the lethal and sub-lethal endpoints were observed with the use of a stereomicroscope according to FET procedure [24]. On the basis of the percentage of lethal defects, LC_50_ values were determined. The incidence of other abnormalities in the development of *Danio rerio* larvae derived from wild-type AB strain as well as their heart rate were also noted. In the above-mentioned strain, the LC_50_ value calculated at 23.04 mg/L and the percentage of other developmental malformations indicate the moderate toxicity of compound towards zebrafish. The incidence of abnormalities increased at a concentration of 16.94 mg/L but mostly remain constant at higher concentrations of newly-synthesized molecule (33.88 mg/L and 84.70 mg/L). Bradycardia after incubation with benzenesulphonyl hydrazone appears also only in case of higher concentrations of the compound tested (33.88 and 84.70 mg/L). Currently, to the best of our knowledge, there is no data in the scientific literature for the study of 4-methylbenzenesulphonyl hydrazone derivatives in the zebrafish model. In order to recall studies using this model, we can resort to substances that have benzene ring with halogen substitution like in the tested *N*-[(3-chloro-4-methoxyphenyl)methylidene]-4-methylbenzenesulphonohydrazide. Such a drug is a nonsteroidal anti-inflammatory drug diclofenac, widely used in clinical practice for many years. Recent acute toxicity studies showed that zebrafish larvae are quite sensitive to diclofenac and the sub-lethal concentration was determined at 3 mg/L [25]. Therefore, further research using mammalian models is planned.

The transgenic *Tg(fli1: EGFP)* zebrafish strain was used as a model for in vivo studies on the anti-angiogenic potential of *N*-[(3-chloro-4-methoxyphenyl)methylidene]-4-methylbenzenesulphonohydrazide. It should be emphasized that there is a translatability of test results between the biology of fish and human vessels, and small particles are able to diffuse into the fish embryo and cause a dose-dependent effect [26]. Other angiogenesis inhibitors (SU5416, TNP470) previously used in mammals have been observed to reduce vessel formation in *Danio rerio* [26]. These substances were administered before the initiation of the angiogenesis process. Moreover, not only inhibitors but also pro-angiogenic factors are effective in the zebrafish model. After administration of human VEGF (vascular endothelial growth factor), an increase in the formation of intersegmental vessels was observed [27].

The experiments in the presented study were performed on embryos which were 6, 12 and 24 h after fertilization (hpf) and they were administered the solutions of the tested compound in concentrations of 1/5, 1/10 or 1/50 LC_50_. After 24-h of incubation, the development of intersegmental vessels of the trunk was analyzed with a fluorescence microscope. The substance inhibited the formation of blood vessels. This effect is only evident when the highest concentration of the compound is used in the 6 hpf and 12 hpf embryo groups. The anti-angiogenic effect of the tested derivative at a dose of 15.81 mg/L as 1/5 LC_50_ is stronger in the 12 hpf embryos and it is comparable to that of sorafenib at a dose of 2.32 mg/L, however in the 6 hpf embryos the above effect occurs more often. It was shown that the tested concentration had an anti-angiogenic effect and at the same time caused no greater mortality than in the negative control group or the presence of any other defects.

To the best of our knowledge, there is a lack of scientific data regarding the study of the anti-angiogenic potential of molecules containing a hydrazone moiety, such as hydrazide-hydrazones or sulphonyl hydrazones, in a zebrafish model. However, the in vitro studies are available. In research published in 2021 by Ihsan Han et al., particular attention was paid to naproxen derivatives with hydrazide-hydrazone moiety [28]. These compounds were tested for their anti-cancer properties on breast cancer cell lines. One of them showed good selectivity for both lines. In addition, molecular modelling of these compounds for the endothelial growth factor receptor VEGFR-2 was performed. It is a type V tyrosine kinase receptor. After VEGF binds to the receptor, a phosphorylation cascade occurs, the effect of which is to increase the migration and proliferation of the vascular endothelium. This receptor is thought to play a role in neoplastic angiogenesis. The possible inhibitory properties of the tested compound on VEGFR-2 were indicated by the IC_50_ values and the free energy of binding, as well as the fact that due to the appropriate chemical structure, the substance fitted into the pocket created by the receptor during docking. What is more, the intermolecular interactions with the receptor were displayed by the hydrazide-hydrazone moiety [28]. In turn, Kassab et al. tested the antiproliferative activity and proapoptotic potential of a series of tolmetin analogues with a structure containing hydrazide-hydrazone moiety in cancer cell lines, i.e., colon cancer cells (HCT-15) [29]. New derivatives were also subjected to molecular modelling which indicated that one of compounds could inhibit VEGFR-2 activity, with an IC_50_ of 0.20 µM. The tested compound also significantly reduced the migration potential of HUVEC, similarly to sunitinib which was used as positive control [29].

The above-mentioned studies indicated plausible anti-angiogenic activity of molecules containing hydrazide-hydrazone moiety [28,29]. While the anti-angiogenic potential of molecule containing the sulphonyl hydrazone moiety, *N*-[(3-chloro-4-methoxyphenyl)methylidene]-4-methylbenzenesulphonohydrazide, synthesized and tested by our research group, was confirmed in in vivo studies carried out on the zebrafish *Tg(fli1: EGFP)* model, which seems to be particularly significant and valuable.

## 4. Materials and Methods

### 4.1. Reagents

All chemicals were purchased from Sigma-Aldrich or Merck KGaA (Darmstadt, Germany). The melting point was determined with the use of Fisher-Johns apparatus (Thermo Fisher Scientific, Inc., Waltham, MA, USA) and it was uncorrected. The ^1^H NMR and ^13^C NMR spectra were recorded on the Bruker Avance 300 apparatus (Bruker Corporation, Ettlingen, Germany) in dimethyl sulfoxide (DMSO-*_d6_*) with the use of tetramethylsilane as the internal standard. The elemental analysis of the obtained molecule was performed using the AMZ 851 CHX analyzer (Gdańsk University of Technology, Gdańsk, Poland). The results of elemental analysis (C, H, N) were within ±0.4% of the calculated values.

### 4.2. The Preparation of N-[(3-chloro-4-methoxyphenyl)methylidene]-4-methylbenzenesulphonohydrazide

**Figure 5:**
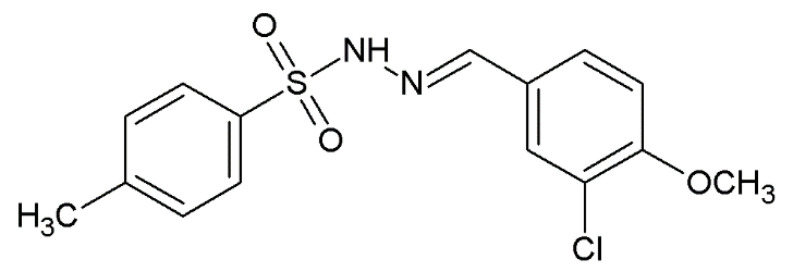
Chemical structure of *N*-[(3-chloro-4-methoxyphenyl)methylidene]-4-methylbenzenesulphonohydrazide.

The synthesis was performed according to procedure described previously by Popiołek et al. [18]. The 4-methylbenzenesulphonohydrazide (0.01 mol) was dissolved in 10 mL 96% ethanol, and then 3-chloro-4-methoxybenzaldehyde (0.011 mol) was added. The mixture was heated under reflux for 3 h. Subsequently, after cooling the solution, the precipitate formed. Then, it was filtered off and recrystallized from ethanol (96%). The physico-chemical and spectral data of obtained derivative is consistent with those reported by Popiołek et al. [18] (Figure 5).

### 4.3. Housing of Zebrafish

Wild-type AB strain and a transgenic *Tg(fli1: EGFP)* strain of zebrafish (*Danio rerio*) were housed in the Experimental Medicine Centre of Medical University of Lublin (Lublin, Poland) where all the experimental procedures on embryos and non-feeding larvae were carried out. Zebrafish strains were kept under recirculating water supply at 26 ± 1 °C and a 10/14 dark–light cycle. The animals were fed with artemia and commercial feed [30]. To obtain the eggs, the traps were placed in the tanks, which had previously been covered with a wire mesh with a mesh size of 2 ± 0.5 mm. This prevents the eggs from being eaten by adult individuals. Traps were set prior to the onset of darkness on the day preceding the test or prior to the lights being turned on the day of the test. Matings, spawning and subsequent fertilization occur within 30 min after switching on the light. The eggs were taken randomly from at least three breeding groups. Two months before spawning, the fish were not subjected to any pharmaceutical treatment. They also showed no signs of disease or infection.

In accordance with the legislation of European Union and Poland, the experiments performed on the earliest life-stages of *Danio rerio* (until 120 hpf), not defined as protected, are not subject to regulations for animal experimentation.

### 4.4. Toxicity Assessment of Test Compound

Fish embryo acute toxicity (FET) tests with both above-mentioned strains of *Danio rerio* were carried out according to the OECD guideline no 236 (2013) [24]. One newly fertilized egg was assigned to each well of 24-well plates. Eight test compound concentrations ranging from 1–100 mg/L were prepared *ex tempore* from a stock solution on each day of exposure. Twenty eggs were individually exposed to each test concentration of molecule and the remaining four eggs were used as internal plate control exposed to E3 medium (i.e., a purified water containing 5 mM NaCl, 0.17 mM KCl, 0.33 mM CaCl_2_H_2_O, 0.33 mM MgCl_2_6 H_2_O and adjusted to pH 7.2). Negative control (twenty-four eggs individually placed into each well filled with 2 mL of E3) as well as solvent control (1% DMSO solution) and positive control (3,4-dichloroaniline at a concentration of 4 mg/L) were also done. FET-test duration was 96 h. Every 24 h, the mortality was recorded on the basis of lethal endpoints as follow: coagulation of fertilized eggs, lack of somite formation, lack of tail detachment and lack of heart beats, using a stereomicroscope (Zeiss, SteREO Discovery.V8, Gottingen, Germany). The half maximal lethal concentration (LC_50_) values were calculated based on the observed mortality of the developing zebrafish exposed to different concentrations of derivative. The LC_50_ value means the concentration of compound causing death of half (50%) of zebrafish embryos/larvae during the duration of the experiment. In case of transgenic *Tg(fli1: EGFP)* strain, FET procedure was only used to establish, on the basis of LC_50_ value, the concentrations needed for ISV angiogenesis assay. Other developmental malformations: edema (heart and yolk), head malformation, spinal and tail deformations, developmental delay defined as lack of hatching success at 96 hpf, were noted among individuals derived from control and treated groups of AB strain of zebrafish. 

### 4.5. Zebrafish Larvae Cardiac Rhythm Measurement

After 96-h exposure of zebrafish embryos/larvae (AB strain) to different concentrations of tested molecule as well as negative and solvent controls, the number of heart beats was visually counted and recorded for each larva during 60 s using a stereomicroscope (Zeiss, SteREO Discovery.V8, Gottingen, Germany) with 40× magnification. To keep the position of the larvae, 3% methylcellulose was used right before recording as the mounting medium.

### 4.6. Angiogenesis Assay

The zebrafish embryos of a transgenic strain *Tg(fli1: EGFP)* with a gene encoding an enhanced green fluorescence protein (EGFP) visualized in the vascular endothelium were used for in vivo intersegmental vessel (ISV) angiogenesis assay [31]. The influence of the newly synthesized derivative on the formation of intersegmental blood vessels between the dorsal longitudinal anastomotic vessel and the dorsal aorta were assessed using a fluorescence microscope. The embryos were treated from 6 to 24 h after fertilization (hpf). The incubation period lasted 24 h [31]. Based on the calculated LC_50_ value, the following concentrations for angiogenesis studies were chosen (as 1/5, 1/10 and 1/50 LC_50_). The above concentrations did not induce the abnormalities or mortality in the FET test. Five 6, 12 or 24 hpf embryos were assigned to each well of 6-well plates. As a positive control, sorafenib -an inhibitor of tyrosine kinases (including VEGFR), was used at two concentrations (1.16 mg/L i.e., 2.5 µM and 2.32 mg/L i.e., 5 µM) [32]. Test concentrations were prepared *ex tempore* from a stock solution on the day of exposure. Twenty-five eggs were exposed to each test concentration of *N*-substituted-4-methylbenzenesulphonyl hydrazone or sorafenib and the remaining five eggs were used as internal plate control exposed to E3 medium (negative controls). 

### 4.7. Statistical Analysis

The LC_50_ value of the tested sulphonyl hydrazone was derived from the concentration-response curve, where the percentage of lethality is plotted versus concentration on a logarithmic scale. Regression analysis was performed with Excel and the above-mentioned value was obtained from linear type of regression analysis. The results of heart rate measurement of 96-h zebrafish larvae were statistically analyzed using one-way ANOVA with Bonferroni correction. The level of significance was *p* <0.05. Research data is presented in the graphs as means ± SEM. The statistical analysis was performed using GraphPad Prism software (version 5.01 for Windows; GraphPad Software Inc., San Diego, CA, USA).

## 5. Conclusions

It may be hypothesized that newly designed compound based on the sulphonyl hydrazone skeleton may demonstrate beneficial anti-angiogenic effects. However, further studies are needed to confirm its ability to inhibit tumor vessel formation using zebrafish or mouse xenograft models. Therefore, in the future, treatment of neoplastic tumors as well as other diseases related to pathological angiogenesis, such as age-related macular degeneration and diabetic retinopathy, can take advantage from plausible anti-angiogenic potential of tested N-substituted-4-methylbenzenesulphonyl hydrazone.

## Data Availability

Data is contained within the article.
